# The Other Side of the Coin: Transesophageal Echocardiography Complications following Cardiac Surgery and Transcatheter Structural Heart Interventions

**DOI:** 10.3390/jcm13154291

**Published:** 2024-07-23

**Authors:** Valeria Maria De Luca, Valeria Cammalleri, Giorgio Antonelli, Sara Bombace, Tobias Friedrich Ruf, Theresa Ann Maria Gößler, Philipp Lurz, Ralph Stephan von Bardeleben, Francesco Grigioni, Gian Paolo Ussia

**Affiliations:** 1 Research Unit of Cardiovascular Science, Università e Fondazione Policlinico Universitario Campus Bio-Medico, Via Alvaro del Portillo, 200, 00128 Roma, Italy; valeriamaria.deluca@unicampus.it (V.M.D.L.);; 2 Department of Cardiology, University Medical Center Mainz, 55131 Mainz, Germany

**Keywords:** TEE, transcatheter, cardiac surgery, complications, injuries, tricuspid regurgitation, mitral regurgitation

## Abstract

Transesophageal echocardiography (TEE) is widely used in cardiac surgery and interventional cardiology and is often an indispensable tool, giving supportive anatomical understanding and smooth guidance in both settings. Despite it being considered safe, fatal complications can commonly occur after a TEE examination in cardiac surgery operating rooms and catheterization laboratories. Currently, there is a lack of awareness of the scale of the problem, as there are only small amounts of data available, mainly derived from the surgical literature. This review summarizes the main predisposing factors for TEE-associated complications (classified as patient and procedure-related) and the main preventive strategies. We aim to apply preventive strategies more broadly, especially to patients at high risk of developing TEE-related serious adverse events.

## 1. Introduction

Transesophageal echocardiography (TEE) was first used in the 1980s and has since become a key instrument that is useful in clinical practice in supporting decision making [1]. Currently, both in outpatient and intensive care settings, as well as for procedural monitoring, TEE provides considerable information [2,3,4,5,6]. Nowadays, intraoperative monitoring during heart surgery is one of the main indications for TEE use. According to international guidelines, TEE should be used in all “open heart” and thoracic aortic surgical procedures and “should be considered” in coronary artery bypass graft surgeries [7]. In addition, elements of peri-interventional imaging, such as percutaneous valve interventions, the correction of intracardiac shunt using occluding devices, and periprosthetic leaks, are major fields of interest in regard to TEE [2,8]. TEE is thought to have a favorable safety profile, but a series of serious TEE-associated complications have been described. Prolonged peri-interventional TEE, usually utilized during intervention monitoring, has been linked to several complications, including upper gastrointestinal (UGI) bleeding, esophageal perforations or esophagogastric erosions, dysphagia, and oropharyngeal lesions [9,10]. Visceral perforations or oral damages are only a couple of the issues that might arise from inserting and manipulating the probe during a TEE. While the upper UGI tract is the site of most of these injuries, other abdominal and respiratory damages have been occasionally documented. Some injuries are more serious and could cause significant morbidities and mortalities, but many minor TEE problems are often undetected [11,12]. Studies conducted during cardiac surgery account for most of the data currently available regarding adverse events related to TEE intraoperative use, with overall rates of TEE-related morbidity ranging from 0.2 to 1.2% [10]. TEE-related injuries during transcatheter procedures are reported with a frequency of 3.3% to 6.1%, suggesting a higher risk of major harm associated with TEE guidance during structural heart intervention than cardiac surgery [12]. Safety is a critical concern given the extensive use of this technique during transcatheter and cardiac surgical interventions. Considering expanding TEE use, we aim to provide useful indications to prevent TEE complications. In addition, we aim to guide the preoperative management of patients at risk of developing TEE-related injuries.

## 2. Definitions

Complications secondary to procedural monitoring with TEE can be classified as gastrointestinal (GI), cardiovascular, and respiratory [11,12,13,14]. A distinction has also emerged between major (life-threatening) complications requiring transfusion or immediate surgical/endoscopic repair and minor (not life-threatening) complications [15].

### 2.1. Gastrointestinal Complications

Associated GI TEE complications include visceral perforations (1), mucosal damage and bleeding (2), and persistent dysphagia (3) [11]. The most common TEE-associated GI complications and their relative frequency in both surgical and interventional settings are shown in Table 1.

Patients with pre-existing, unrecognized esophageal injuries are most at risk of suffering these complications. Erosion-causing esophageal reflux, inflammation, tumors, achalasia, diverticula, hiatal hernia, history of radiation therapy and arthritic changes in the cervical spine appear to be the predisposing factors for this type of injury [21,22,23].

The esophagus can be perforated (1) in four different segments: the pharyngeal (11.8%), cervical (20.6%), intrathoracic (54.3%), and abdominal (11.8%) sections [24,25]. The esophageal wall has an inherent weakness brought on by fibers originating from the pharyngeal constrictor and the cricopharyngeal muscles, making the hypopharynx and upper esophagus the areas susceptible to perforation [26]. For the cervical esophageal portion perforation, the most frequent cause is probe intubation. In fact, it is feasible for the probe to repeatedly glide into a Killian−Jamieson or Zenker’s diverticulum during esophageal intubation [27]. Depending on its size and depth, the probe will be introduced into the diverticulum at a varied length until resistance is felt. The risk of morbidity increases after vigorous insertion because it is easy to harm an esophageal diverticulum [21,22]. Regarding damage in the thoracic esophageal and gastric portions, the most prevalent cause is prolonged forced contact with the probe [11]. The TEE probe can occasionally generate pressures of up to 60 mmHg, which can lead to compression and damage, although pressures of less than 17 mmHg are potentially unsafe [28]. In addition, there are isolated reports of splenic rupture after transgastric TEE in patients with splenomegaly due to traction of the gastrosplenic ligament containing the short gastric vessel [29,30].

Bleeding (2) after a TEE in the operating room (OR) or in the cath lab is usually superficial and originates from the hypopharynx or is the result of tooth injuries or tonsil erosions, but it can also be more severe and dangerous. Risk factors for UGI bleeding include gastroesophageal varices, the use of vasoactive drugs, a history of ulcerative processes, and the lack of proton pump inhibitors (PPIs) at the hospital admission before procedures. In addition, contact with the probe and viscera, and the resulting heating of the mucosa, leads to heat-related damage [31]. Moreover, it has been previously shown that Mallory–Weiss tears in conjunction with antecedent TEE frequently result in catastrophic hemorrhage [32,33,34]. Dysphagia (3) is also independently associated with perioperative TEE morbidity [35]. The pharyngoesophageal tissue and the laryngeal nerve may be locally compressed during the insertion technique, which could be one of the causes of dysphagia. Female patients are more likely to suffer from laryngeal nerve palsy than male patients due to the narrower laryngeal anatomy in females. In 90% of instances, dysphagia was linked to pulmonary aspiration, which increased the need for tracheostomies and prolonged hospital stays. Advanced age, the length of surgical intubation, and perioperative TEE examination are risk factors for this complication [19,36,37].

### 2.2. Cardiovascular and Respiratory Complications

Cardiovascular complications secondary to TEE during procedures are due to vagal reflexes or, conversely, sympathetic stimulation after probe insertion [11]. Although older patients suffering from heart failure are more vulnerable in general, anesthesia may also exacerbate hypotension in these individuals [38]. Arrhythmic complications such as advanced atrioventricular blocks, non-sustained ventricular arrhythmias, and paroxysmal atrial fibrillation, may eventually occur [21].

Inserting, removing, or moving the probe can also affect the position of a correctly positioned endotracheal tube. This is especially true for pediatric patients in whom ex-tubation, displacement into a large bronchus, pilot cuff injury, and a 1–2% incidence of severe airway obstruction have been identified [39].

However, rare cases of airway obstruction caused by similar mechanisms have been reported in adults [40]. The compression by the esophageal probe may be indicated by decreased oxygen saturation, elevated ventilation pressure, and altered end-tidal CO_2_ breathing patterns; therefore, these signs should be continuously monitored [41].

## 3. TEE Complications after Cardiac Surgery

TEE is a very useful diagnostic tool for monitoring heart operations. In the OR, TEE can appraise real-time biventricular functions, the choice of valve repair or replacement, the detection of possible complications, and the result of surgery. However, TEE itself can occasionally result in esophageal, gastric, and oropharyngeal injuries. Local pressure effects, vascular insufficiency, local tissue thermal injury, and poor mucosal blood supply can contribute to the mechanism of TEE probe damage during cardiac surgery [16].

In cardiac surgery, patient and procedure-related characteristics are recognized risk factors in regard to TEE complications. Elderly and immunocompromised patients, as well as subjects with previous cerebrovascular accidents and GI diseases, are among the patient-related factors that raise the risk [12].

Nowadays, however, the most fragile and vulnerable patients, the elderly and those with more comorbidities, are usually referred for transcatheter interventions [42]. This may explain, at least in part, the lower rate of TEE-related complications in patients undergoing cardiac surgery compared to transcatheter interventions. Thus, patient-related factors account for a smaller proportion of the causes of TEE damage in the surgical population.

The Heart Team plays a crucial role in this setting, guiding treatment decisions by integrating clinical, anatomical, and procedural characteristics beyond standard scores, as supported by current data [42]. However, it is important to weigh the benefits and drawbacks of surgery vs. interventional cardiology and emphasize the potential risks associated with each strategy to provide more informed decisions on which procedure is best for a given patient. Indeed, patients with more comorbidities and aged over 75 years are generally referred for interventional procedures.

Surgery-related factors include the type of surgery other than coronary artery bypass graft (CABG) surgery, length of intubation, time undergoing TEE, increased extracorporeal circulation time, return to OR for revision, and higher Activated Clotting Time (ACT) during operation [12]. Moreover, given the increased temperature attained and the greater dimensions (16.6 mm in width versus 14.9 mm) compared to a standard adult 2D TEE probe, the incidence of TEE complications may be higher with a real-time three-dimensional TEE (RT-3D TEE) probe [43]. The recent introduction of mini 3D probes could resolve this issue in the future [44].

Therefore, intervention-related factors represent the main events causing GI damage in the surgical population. A TEE evaluation of the postoperative findings is necessary for certain surgeries, such as valve replacement or repair, which usually take longer than CABGs. In these cases, damage may have been caused by significant anteflexion of the probe tip for long periods and by the pressure that the probe applied to the mucosa to achieve transgastric and deep transgastric projections [17].

In addition, the probe is usually left in place for the whole operation. It is frequently in a stationary position for hours at a time, causing extended contact with esophageal mucosa areas with consequent heating damage [24]. Further factors that may increase the mucosa vulnerability to pressure necrosis and ischemia include anticoagulation during the surgical phase, hypothermia, and reduced blood flow [11].

Kallmeyer et al. examined 7200 cardiac surgery patients who had intraoperative TEE at a single center and reported a TEE-related morbidity of 0.2% [18]. The most frequent complication was odynophagia requiring further investigation, with esophagogastroduodenoscopy (EGD) occurring in 0.1% of the patients. Of these individuals, five (0.07%) had dysphagia. Two cases (0.03%) presented acute post-surgical hemorrhage, one of them due to Mallory–Weiss syndrome and one case being due to esophageal perforation (0.01%) in the thoracic tract requiring repair [18]. During TEE probe manipulation, 0.03% of patients experienced an unintentional advancement of the endotracheal tube into the right mainstem bronchus, resulting in respiratory adverse effects due to arterial desaturation [18].

Overall, 6 (1.2%) out of 516 participants who underwent TEE during cardiac surgery in a study by Lennon et al. [16] had significant GI problems. Two patients (0.4%) had stomach perforations, one patient (0.2%) had a gastric ulcer, and three patients (0.6%) had esophageal or gastric tears needing laparotomies, endoscopic procedures, or transfusion care. In the cardiac surgery group, the thoracic portion of the esophagus was the most susceptible to perforation (73%) [24].

In a study by Hulyalkar et al. that included both retrospective and prospective data, overt UGI bleeding occurred in 5 of 241 patients (2.1%) after cardiac surgery with TEE [20]. Furthermore, from the case studies of Kiran et al., up to 27.5% of candidates for cardiac surgery had a difficult insertion of the probe that required additional maneuvers [43]. According to McSweeney et al., 1.2% of the patients in their cohort who had undergone CABG experienced postoperative GI bleeding, and this condition was linked to the highest risk of stroke, renal failure, and in-hospital mortality [45].

In a prospective observational piece of research involving 869 patients undergoing heart surgery, Hogue et al. found that 4% of patients had dysphagia [19]. Among them, 34 (4%) underwent postoperative barium roentgenography, showing swallowing impairment linked to TEE [19]. Furthermore, the significant correlation found between swallowing difficulties and tracheostomy, postoperative pneumonia, and prolonged hospitalization in the intensive care unit underlined the seriousness of this surgical complication [19].

Accordingly, the currently available information suggests that, in skilled hands, TEE application in cardiac surgery is both highly effective and safe. However, most of the available data are retrospective, and EGD is not routinely performed postoperatively, so minor damage may not be detected. In addition, early detection and treatment of TEE complications are crucial to achieving the best results and reducing mortality. It is important to be aware that TEE-related damage may not only happen in the immediate postoperative period (early presentation < 24 h) but also after a few days (late presentation > 24 h).

In the days following the procedure, an adequate history and detailed physical examination may be useful to detect clinically manifest events, while routine EGD is not recommended. The use of chest radiographs after the procedure may improve the timely detection of serious TEE-related complications. Hasami et al. reported a case of mid-thoracic esophageal perforation that was discovered by air-fluid configuration on a chest X-ray up to four days after surgical aortic valve replacement plus CABG surgery in a completely asymptomatic patient [46]. Akao et al. reported a case of a giant esophageal submucosal hematoma diagnosed by a chest CT scan on the fourth postoperative day in an 81-year-old man who underwent mitral valve surgery, probably as a result of manipulation with the TEE probe [47].

Consequently, investigating the occurrence of symptoms and signs of GI damage in patients undergoing TEE postoperatively is mandatory.

## 4. TEE Complications after Cardiac Transcatheter Procedures

**Figure 1:**
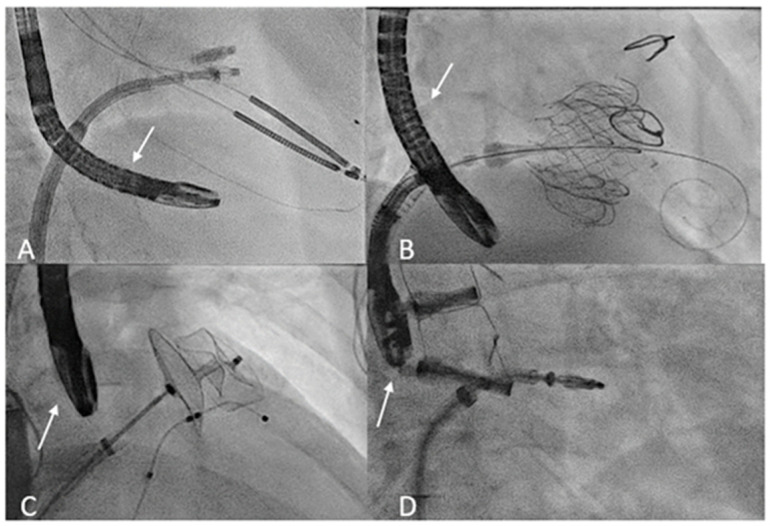
Transoesophageal probe position (arrow) during transcatheter interventions. (**A**) The fluoroscopy shows the transgastric probe position during T-TEER. (**B**) Deep esophageal view used during tricuspid valve replacement. (**C**) Mid esophageal probe position during LAA occlusion. (**D**) Mid esophageal probe position used during M-TEER. T-TEER: Tricuspid Transcatheter edge-to-edge repair; LAA: left atrial appendage; M-TEER: Mitral Transcatheter edge-to-edge repair.

In interventional cardiology, TEE is often used for preoperative cardiac data evaluation, but procedure guidance and outcome assessment are also of crucial importance. In particular, TEE is an irreplaceable tool in terms of obtaining an optimal result. TEE used to guide catheter-based procedures involves constant manipulation of the probe during the interventions, in contrast to the TEE sequence utilized in an OR (Figure 1). In addition, patients undergoing percutaneous procedures tend to be sicker than patients undergoing surgery and may therefore be more susceptible to complications associated with intraprocedural TEE [42]. As such, there is a higher incidence of TEE-related problems in this scenario than in cardiac surgery populations [10].

Also in this setting, predisposing factors complicating TEE can be distinguished as patient-related or procedure-related.

In a study by Freitas-Ferraz et al., in enrolling 1251 patients undergoing cardiac interventional procedures with an intraoperative TEE, a low body mass index (BMI), a prior history of GI bleeding, and the use of chronic corticosteroids and immunosuppressive drugs were significantly associated with an increased risk of TEE-related complications [10]. The low weight may be a marker of frailty, which has been linked to an increased risk of postoperative complications and in-hospital mortality [48]. Conversely, larger patients can accommodate the relatively large probe more [49]. Evidence for this has been demonstrated in the pediatric population [39,50]. In addition, patients with friable mucosal tissue from the underlying disease or the use of long-term corticosteroids are at higher risk of developing TEE-related injuries [10,51]. Similarly, anticoagulants increase the risk of GI bleeding. Natale F et al. recommended high vigilance for anticoagulated patients, with careful consideration of the risk-benefit ratio of the intervention, especially in patients with an INR > 5, even in urgent situations where relative contraindications are often overlooked [52]. Injuries to the gastrointestinal tract (erosions, ulcerations, or subclinical bleeding) also occur frequently in patients treated with antiplatelet agents [53]. It can be challenging to manage patients on antithrombotic medication who are having endoscopic procedures, and therapy strategies frequently need to be customized [54].

The most significant procedure-related issue is the amount of time spent undergoing TEE. It also emerged that the time undergoing TEE was an independent predictor of serious adverse events, with an odds ratio of 1.13 for every ten-minute increase [55].

In addition, the handling of probes and the resulting risk of complications varies depending on the procedure type. Low-risk cohorts—like patients undergoing transcatheter aortic valve replacement (TAVR)—have a risk of up to 0.9% in regard to associated TEE complications. Emphasizing that low-risk procedures are not zero-risk is crucial. Ismail et al. [56] reported a rare case of esophageal perforation in patients undergoing TAVR under TEE guidance [56]. This risk is between 0.03% and 0.09% [17,55] but has a significant mortality rate of about 16% [57]. For high-risk cohorts, like those undergoing atrioventricular valve repair or replacement, left atrial appendage occlusion (LAAO), and paravalvular leak closure (PVLc), the risk reaches 6.1% [5,10]. In detail, 0.5% esophageal perforations, 1.4% esophageal tears, 0.9% wall hematomas, and 0.5% bleeding requiring transfusion are reported [10]. Time undergoing TEE and continuous adjustment maneuvers result in an increased risk of damage. Thus, in contrast to TAVR, active guidance and image optimization are necessary during every step of the high-risk group procedures. Actually, fluoroscopic guidance is usually sufficient for valve deployment in TAVR patients, and a complementary TTE may be useful to rule out any serious complications [8]. In a study by Freitas-Ferraz et al., EGD performed after the procedure and before extubation showed new TEE damage in 86% of patients after interventional procedures. In particular, 40% of these were complex lesions, such as 28% of esophageal hematomas, 2% of soft palate hematomas, 24% of esophagus lacerations, and 2% of stomach lacerations, whereas 22% had persistent odynophagia or dysphagia [55]. Moreover, in a cohort of 12,043 adults undergoing TEE-guided structural interventional procedures, 3.6% experienced major TEE complications [58]. Furthermore, among patients undergoing TAVR, transcatheter edge-to-edge mitral valve repair (M-TEER), percutaneous LAAO, or a mitral/aortic PVLc, those after M-TEER are the most prone to complications [10]. This result confirms the critical role of the TEE guidance in the intricate process of edge-to-edge mitral valve repair.

As for patients undergoing tricuspid valve edge-to-edge repair (T-TEER), the complication rate is up to 3.1% [15]. Specifically, 1.6% esophageal perforations and 1.6% gastric injuries were described in the study by Hellhammer et al. in which 64 patients were retrospectively enrolled [15]. A recent publication by our group reports the absence of clinically manifest major or minor TEE-related complications in a small group of 53 patients who underwent T-TEER who were prospectively evaluated [59]. However, data on TEE complications after transcatheter tricuspid valve intervention are lacking, and the results could be underestimated. Because of its anterior location, the tricuspid valve is challenging to see in regard to TEE. It is necessary to precisely visualize the leaflets during the procedure by switching between transgastric and mid/deep esophageal views. This requires prolonged anteflexion of the probe, which may result in damage [60]. Furthermore, mainly in repair and replacement procedures, extensive use of 3D images with live multiview is often requested by interventional cardiologists. The use of 3D should make the procedures faster for a better recognition of anatomical relationships with the devices used and thus decrease the time undergoing TEE. On the other hand, 3D probes are more likely to sustain thermal damage since they reach higher temperatures and have a larger size when compared to the 2D probes. All this could translate into an increased risk of procedural complications.

Further investigations are required to determine the severity of the issue in the group of patients who underwent a tricuspid valve replacement or repair. Therefore, as with the data available for subjects undergoing cardiac surgery, and as with the transcatheter setting, the studies are often retrospective and without post-procedure invasive endoscopy. The more recent procedures—like those on the tricuspid valve, for which there are insufficient data—are another constraint in this specific scenario.

Thus, patients with clinical symptoms are those with relevant complications that we are able to detect. Minor injuries may go undetected. Accordingly, the problem of TEE-related complications may be more extensive than expected, especially in the transcatheter group.

## 5. Prevention

Although TEE-related complications are not as common, in some settings (such as cardiac surgery and interventional cardiology) they might still cause issues such as major bleeding and organ perforation. In particular, TEE-related problems can lengthen average hospital stays and increase mortality to 28.5% [24]. For these reasons, it is important to prevent associated TEE complications, especially in these scenarios [59]. Since predisposing factors can be distinguished into being patient-associated and procedure-associated, preventive strategies can also follow this distinction.

Regarding patient-related strategies, it is important to estimate the individual risk of TEE complications. Investigations into dysphagia, GI diseases, known diverticula, previous endoscopies, bariatric surgery, and/or chest radiotherapy should be considered. It is also important to consider medications taken by the patient that could be associated with a high risk of injury, such as corticosteroids, immunosuppressants, antiplatelets, or anticoagulants. An assessment of neck mobility, stability, and arthritic changes, an analysis of the airways, and an evaluation of teeth mobility are all required components of the examination [11]. A preventive strategy is to schedule a gastroenterology consultation and possible endoscopy before the procedure in patients who may already have gastroesophageal lesions [12]. Therefore, in patients with known esophageal or gastric injuries, dysphagia and/or a history of severe upper digestive tract bleeding and/or major upper digestive tract surgery, a more thorough specialist gastroenterologic evaluation and possibly an EGD should be performed prior to cardiac surgery or transcatheter surgery requiring extensive use of TEE. In this setting, endoscopy is a useful tool for assessing the presence of absolute contraindications to performing TEE.

In patients with a history of bariatric surgery, TEE is perceived as a relative or absolute contraindication [61]. A retrospective study of Kelava M. et al. showed a 91% rate of TEE performed in a cohort of 282 patients with previous bariatric surgery. In the majority of patients, examinations were limited to the upper or midesophageal views and no TEE-related complications were observed [61].

In an effort to reduce GI issues, it is critical to evaluate the potential benefits of a TEE examination against the specific dangers when contemplating the procedure for a patient with a relative contraindication.

If the risk of complications is high and TEE guidance for the surgery is not essential (such as for CABG), TEE could be avoided or the TEE examination limited to a preoperative study, e.g., when excluding a significant valve lesion in a patient in whom CABG is planned. In addition, an intraoperative epicardial ultrasound can be used successfully in selected patients [61]

**Figure 2:**
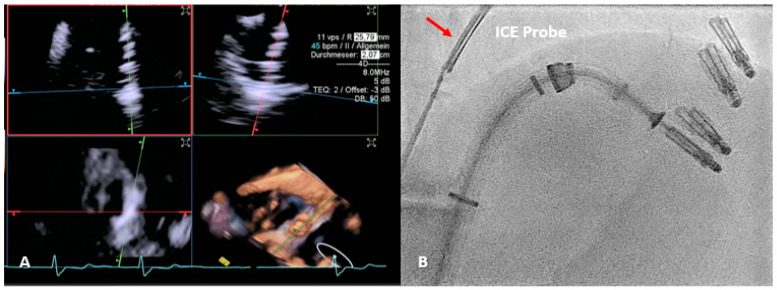
ICE application during T-TEER. (**A**) illustrates the live multiplanar reconstruction process used to align the TriClip (Abbott Vascular, Santa Clara, CA, USA) device on the tricuspid valve, for grasping the anterior and septal leaflets. In (**B**), the fluoroscopic position of the intracardiac probe can be observed (red arrow). The proximity of the probe to the device allows us to overcome shadows by obtaining high-quality images. T-TEER: Tricuspid Transcatheter edge-to-edge repair; ICE: intracardiac echocardiography.

However, TEE guiding is often not avoidable, such as during valve repair or replacement interventions [24]. This is frequently not feasible in the transcatheter context, so alternative approaches must be considered. Specifically, the mini 3D TEE probe can be used in these circumstances [44]. Patients suffering from esophageal stricture can also benefit from this option. For optimal intraprocedural imaging, intracardiac echocardiography (ICE) may also be a useful tool (Figure 2). Due to its ability to produce high-resolution near-field imaging, ICE can overcome TEE limits, such as acoustic shadowing, and avoid GI or respiratory complications. However, the high expense of this technology severely restricts the application of ICE in routine clinical practice [62,63,64].

**Figure 3:**
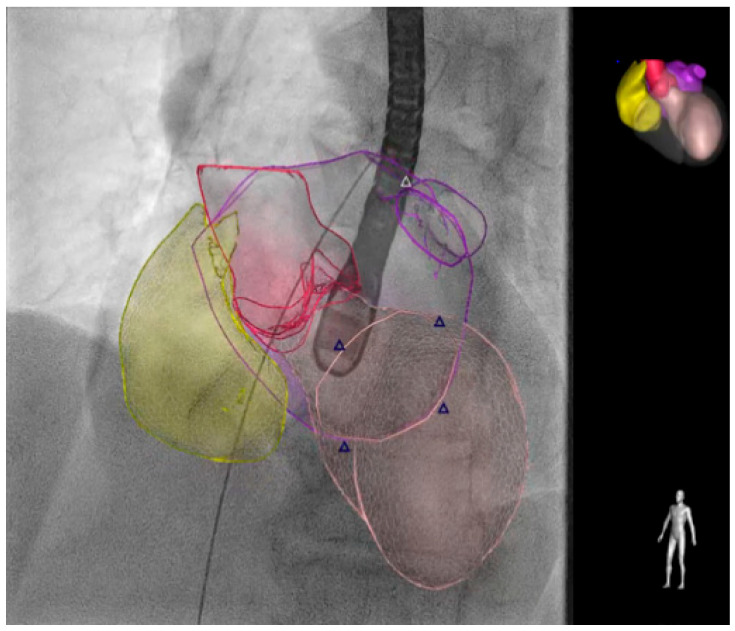
The EchoNavigator^®^ (Philips Medical System, Andover, MA, USA) live image guidance. The smart fusion technology simplifies visualization by fusing real-time X-rays and live echo. EchoNavigator may give interventionalists a supportive anatomical understanding and smooth guidance during structural heart disease interventions to reduce the time undergoing TEE. The different colors distinguish various cardiac structures, with the triangles indicating the position of the mitral valve. The right atrium is shown in yellow, the aorta in magenta, the left ventricle in pink, and the left atrium in purple as indicated in the cartoon in the top right. TEE: transesophageal echocardiography.

When alternatives are unavailable, fusion imaging (Figure 3) and transthoracic echocardiography may be used as an alternative to traditional TEE [65,66].

It is also crucial to assess the patient’s drug treatment ahead of interventions and optimize therapy in order to prevent GI damage. Taking PPIs on hospital admission can decrease the overall risk of GI complications. Accordingly, initiation of PPI therapy at the time of hospitalization of patients scheduled for cardiac surgery or transcatheter intervention could be recommended to prevent TEE-related complications [59]. In selected patients, intraprocedural infusion of PPIs could also be considered. Furthermore, appropriate management of anticoagulation therapy is cardinal. It is recommended that patients discontinue anticoagulants before surgeries based on the surgery-related bleeding risk and the patient’s condition [67]. High watchfulness for anticoagulated patients, especially those with an INR value > 5, is strongly recommended [52].

Regarding procedure-related strategies, it is imperative that the imaging operator first examines the condition of the probe and checks for any wounds that may cause thermal damage and overheating. To guarantee probe flexibility and avoid esophageal tears caused by rigid tubes, the control system needs to be unlocked constantly [68]. After placement of the mouthguard, the probe should be gently introduced into the esophagus. Adequate probe lubrication can limit friction damage. If resistance is encountered, it is important to avoid forceful manipulation. In this case, the jaw-thrust technique may be helpful. Placement under laryngoscopic guidance also can help reduce hypopharynx and cervical esophagus damage [11,59]. Furthermore, a thorough assessment of vital signs and oxygen saturation can suggest the possibility of endotracheal tube displacement during esophageal intubation. Avoiding unnecessary manipulation after positioning the TEE probe can help to limit adverse effects. Moreover, anatomical markers of the probe position under fluoroscopy can help identify the correct placement and prevent over-probe manipulation when getting an acceptable alignment between the probe and the cardiac structures is difficult (Figure 1).

Additionally, to prevent overheating and thermal harm to the esophageal mucosa, it is mandatory to freeze the probe when not in use [12]. An additional strategy could be dynamic TEE guidance of the procedure, avoiding prolonged flexion maneuvers at the same mucosal site as often as possible and alternating different projections. Complications are more likely when the probe position is blocked in an attempt to obtain higher-quality pictures. Therefore, to enable proper directing, this method should only be applied in circumstances of high acoustic impedance; otherwise, this routine attitude should be avoided. Furthermore, excessive manipulation can be minimized by adjusting the parameters based on the lowest output intensity required to ensure satisfactory image quality [55]. It is imperative to employ techniques to enhance the quality of the image, such as adjustments to the 2D depth, sector size, focus and gain, and zoom, which allow the operator to maximize image quality while reducing emission intensity. By applying the ALARA (As Low As Reasonably Achievable) principle, it is possible to obtain useful diagnostic information with minimal risk to the patient, using the lowest level of ultrasound exposure that is reasonably achievable [69].

A further concern with TEE-related complications is the risk of infection due to the pathogen transmission among sequential patients. This indicates a need for dedicated protocols for the patient and the healthcare team’s protection. The use of probe covers could at least partially reduce the infection risk [70]. However, their use could be impractical during interventional procedures, limiting the echocardiographic windows. In addition, undetected small cracks have been correlated with poor sterilization and increased risk of infections, such as nosocomial pneumonia [71].

Time spent undergoing TEE is a major risk factor in regard to problems [10]. Thus, the interventional cardiologist/surgeon and the imager’s skills are crucial. Adequate preoperative planning can allow the interventional strategy to be chosen in advance, reducing the time of procedures. In addition, 3D analysis should be limited to key steps to reduce the probe overheating. Further 3D assessments, when possible, should be deferred to post-processing. Early detection of any complications may also limit the consequences. Therefore, careful monitoring of hemoglobin values and a follow-up chest X-ray should be performed [10,72,73,74]. On the contrary, considering that most patients have self-limiting symptoms or are asymptomatic, as well as that most problems can be managed conservatively, a routine endoscopic post-procedural investigation would not be cost-effective and should not be advised for every patient [55].

## 6. Conclusions

Even if TEE guidance is required in therapeutic settings, it is appropriate to be aware of the potential consequences. Unfortunately, there is a dearth of information about the actual incidence of events, particularly for more recent interventions. It is critical to understand potential TEE-related consequences and put preventive measures in place to lower deaths and morbidities (summarized in Table 2).

## 7. Future Directions

TEE-guided treatments provide many advantages; however, significant hazards need to be considered, especially as it is to be expected that the use of TEE will increase in the coming decades. The inclusion of anatomical information, mini-TEE probes, ICE, and fusion approaches could improve some of the disadvantages of conventional TEE. Enhanced adoption of alternative techniques and appropriate risk assessment should help reduce the frequency of TEE-related complications.

## Figures and Tables

**Table 1:** Frequency of the most common TEE-related complications in cardiac surgery and interventional cardiology setting (adapted with permission from [10,15,16,17,18,19,20]).

TEE-Related Complications	Cardiac Surgery	Interventional Cardiology
Dysphagia/odynophagia	0.1–4%	22%
GI Perforations	0.01–0.4%	0.03–1.6%
Esophageal/gastric injuries	0.6%	1.4–28%
Bleeding	0.03–2.1%	0.5%

TEE: transesophageal echocardiography; GI: gastrointestinal.

**Table 2:** Recommendations for the prevention of major TEE complications.

TEE-Related Complications	Risk Factors	Contraindications	Preventive Strategies
Dysphagia/odynophagia	Time spent undergoing TEE Careless intubation	-	Gentle intubation; avoid overheating of the probe.
GI Perforations	Previous perforations Ulcers	Known untreated gastric lesions	Preventive EGD in high-risk patients. Consider alternative interventional imaging.
Esophageal/gastric injuries	Previous Bariatric Surgery Previous Radiotherapy Known diverticula	Tight esophageal stricture	Avoid probe lock and freeze when not in use. Apply the ALARA principle.
Bleeding	Antiplatelets Anticoagulants Thrombocytopenia	Active bleeding unclear genesis	Careful evaluation of anticoagulant and antiplatelet therapy before the procedure.
Infection	Frailty Immunosuppression Probe cracks	-	Use protective covers in selected patients. Watchful probe examination

TEE: transesophageal echocardiography; GI: gastrointestinal; EGD: esophagogastroduodenoscopy; ALARA: As Low As Reasonably Achievable.

## Data Availability

Not applicable.
